# Intraoperative Photodiagnosis for Malignant Glioma Using Photosensitizer Talaporfin Sodium

**DOI:** 10.3389/fsurg.2019.00012

**Published:** 2019-03-21

**Authors:** Jiro Akimoto, Shinjiro Fukami, Megumi Ichikawa, Awad Mohamed, Michihiro Kohno

**Affiliations:** ^1^Department of Neurosurgery, Tokyo Medical University, Tokyo, Japan; ^2^Department of Neurosurgery, Kohsei Chuo General Hospital, Tokyo, Japan; ^3^Department of Neurosurgery, Sohag University, Sohag, Egypt

**Keywords:** malignant glioma, photodiagnosis, talaporfin sodium, fluorescence guided resection, tissue concentration

## Abstract

**Objective:** The aim of this study was to demonstrate the clinical feasibility of intraoperative photodiagnosis (PD) of malignant brain tumor using talaporfin sodium (TPS), which is an agent used in photodynamic therapy (PDT) for cancers.

**Methods:** Forty-seven patients diagnosed with malignant gliomas by preoperative imaging (42 patients with gliomas and 5 patients with other brain tumors) received an intravenous injection of TPS at 40 mg/m^2^ 24 h before resection. During surgery, these patients were irradiated with diode laser light at 664 nm, and tumor fluorescence was observed. The fluorescence intensity was visually rated on a 3-point rating scale [strong fluorescence, weak fluorescence and no fluorescence]. TPS concentrations in 124 samples from 47 cases were measured by HPLC (High performance liquid chromatography).

**Results:** The fluorescence intensity was confirmed to be weak in all patients with Grade II gliomas and strong in almost all patients with Grade III or IV gliomas, reflecting the histological grade of malignancy. In patients with non-glioma brain tumors except for 1 patient with a metastatic brain tumor, the fluorescence intensity was strong. The mean TPS concentration in tissues was 1.62 μg/g for strong fluorescence areas, 0.67 μg/g for weak fluorescence areas and 0.19 μg/g for no fluorescence areas.

**Conclusions:** Establishment of an appropriate fluorescence observation system enabled fluorescence-guided resection of malignant brain tumors using TPS, and the fluorescence intensity of tumors correlated with the TPS concentrations in tissues. These results suggest that TPS is a useful photosensitizer for both intraoperative fluorescence diagnosis and photodynamic therapy.

## Introduction

The use of photosensitizers (PSs) for malignant gliomas has been aimed mainly at tumor control by photodynamic therapy (PDT). In 1980, a clinical report related to PDT, in which patients with gliomas underwent helium-neon laser irradiation after the administration of Photofrin II, was submitted by Perria et al. (1) Since this report, clinical studies of PDT with dye laser irradiation mainly using hematoporphyrin derivatives (HpDs) have been reported (2–5). The main mechanism of PDT is based on the generation of cytotoxic singlet oxygen in tissues under aerobic conditions, as a result of a photochemical reaction induced by the administration of a non-toxic PS in combination with the irradiation of an excitation laser specific to the PS. The cytotoxic effects of this singlet oxygen cause damage to tumor cells and newly formed blood vessels (6, 7).

After administered to a patient, PS is transformed from the ground state to the excited state by laser irradiation, and immediately returns to the ground state with emitting fluorescence. In 2000, Stummer et al. established a methodology called fluorescence-guided resection (FGR) where tumors are resected using an intraoperative photodiagnosis (PD) of malignant gliomas with the fluorescence as a guide (8, 9). The FGR was acclaimed as a breakthrough methodology where PS is used for purposes other than the original intended use in PDT. A phase III study conducted thereafter proved that the improved extent of resection achieved by FGR would prolong progression free survival (PFS), and FGR has gained recognition as a methodology with a high level of evidence (10–12). The PS they used was 5-aminolevulinic acid (5-ALA), which was thereafter approved as an agent for intraoperative localization of malignant gliomas by the European Medicines Agency in 2007, in Korea in 2011 and also in Japan in 2013. Theoretically, 5-ALA can also be applied to PDT, and basic research and clinical research of PDT using 5-ALA in malignant gliomas have been reported. To date, however, no evidence for the usefulness of 5-ALA in PDT comparable to that in PD has been reported (13, 14).

The authors reported the safety and efficacy of PDT using an HpD, talaporfin sodium (TPS), which is a second-generation photosensitizer, by conducting an *in vitro* study in human glioma cells (15), an *in vivo* study in an experimental model of transplanted C6 glioblastoma in rats (16, 17), and furthermore clinical research including investigator-initiated studies in patients with malignant gliomas (18, 19). Partially because TPS had already been approved as an agent for PDT in patients with early stage lung cancer (20), TPS and an excitation diode laser device specific to TPS were approved for health insurance reimbursement in Japan in 2013 as an agent for PDT in patients with primary malignant brain tumors.

In our previous clinical research on PDT, all patients preoperatively predicted to have malignant gliomas received TPS and underwent FGR (18). Then, PDT was performed only for patients suggested to have residual tumor based on PD. In other words, we found TPS to be useful for both PD and PDT and consider TPS an ideal PS to date in the treatment of malignant brain tumors. In this article, we report the usefulness of PD in the treatment of primary malignant brain tumors with TPS administration and excitation laser irradiation, by presenting actual cases. We also report the correlation between PD, which involves gross assessment, and actual TPS concentrations in brain tumor tissues.

## Materials and Methods

### Talaporfin Sodium and Diode Laser

**Figure 1:**
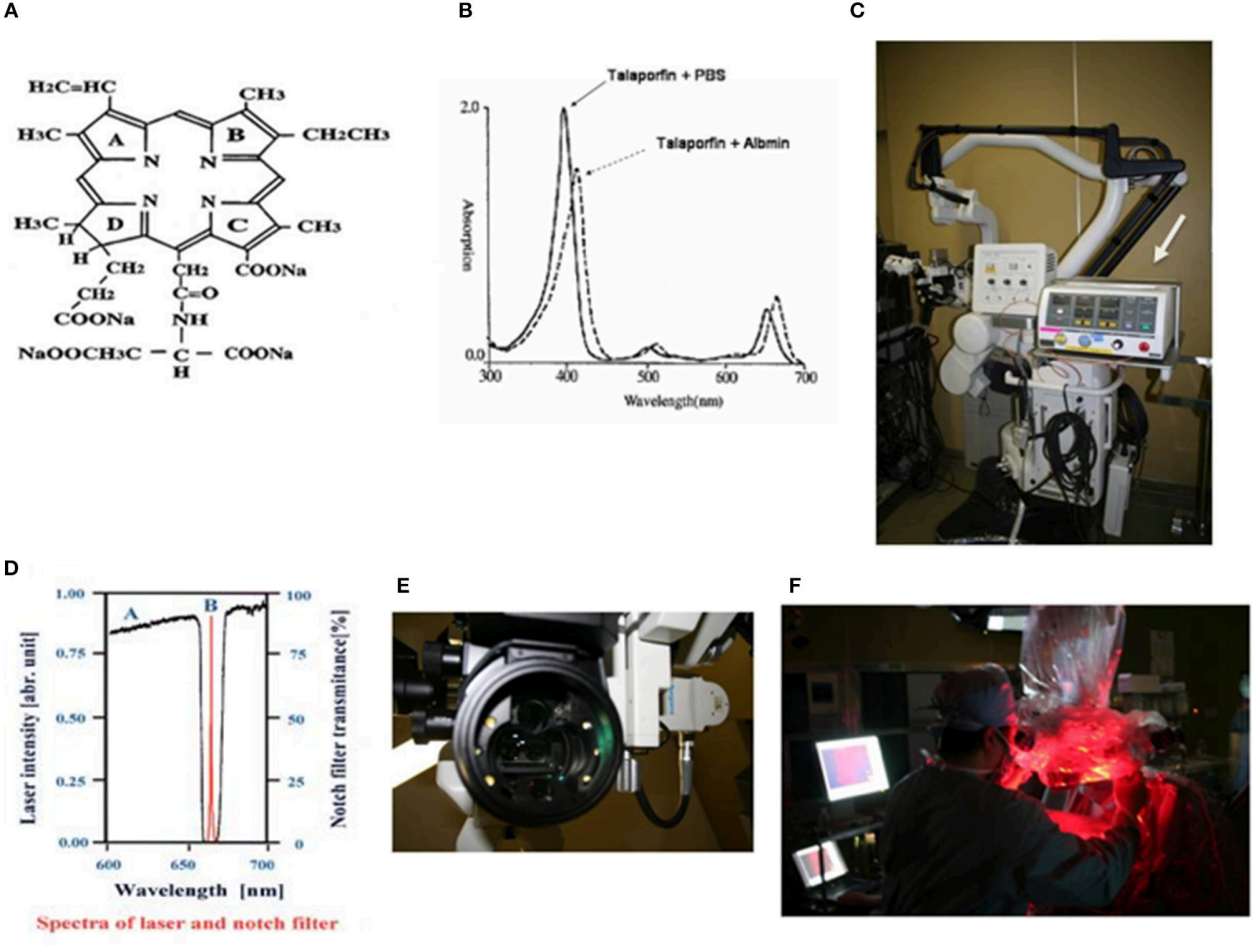
**(A)** Chemical structure of talaporfin sodium (mono-L-aspartyl chlorine e6, NPe6). **(B)** Absorption spectrum of talaporfin sodium and change in absorption wavelength following conjugation with albumin (solid line: talaporfin sodium and phosphate buffer solution, dotted line: talaporfin sodium conjugated with albumin). Talaporfin sodium has absorption peaks in the Soret band (398 nm) and Q bands (502, 530, 620, and 654 nm) in pH 7.4 phosphate buffer solution (PBS). When it conjugates with albumin, its absorption band wavelength becomes ~10 nm longer (bathochromic shift). **(C)** MM80® surgical microscope (Mitaka Kohki Co., Ltd.) equipped with a compact diode laser (PD Laser®, Panasonic Health Care Ltd., arrow). **(D)** A very narrow notch filter (Nitto Optical Co. Ltd.) that can cut out light excited at 664 nm and can capture fluorescence emitted at 672 nm is mounted in a surgical microscope. **(E)** Six white LEDs (Nichia Corporation) are mounted as light sources in the applicator at the objective lens side of the microscope, in order to illuminate the brain surface during laser irradiation. **(F)** From the surgical microscope, the diode laser irradiates the surgical field.

Talaporfin sodium (TPS: Laserphyrin®, Meiji Seika Pharma Co., Ltd., Tokyo, Japan) is a photosensitizer, a hydrophilic compound manufactured by coupling aspartic acid and chlorine, utilized in PDT, which was approved for use in Japan in 2003 as treatment for early-stage lung cancer, in combination with a diode laser (PD Laser®, Panasonic HealthCare Co., Ltd., Ehime, Japan) at a wavelength of 664 nm (Figures 1A,B). TPS is a second-generation photosensitizer that is more quickly excreted from the body than the first-generation porfimer sodium (Photofrin®, Pfizer Japan Inc., Tokyo, Japan). It is characterized by rapid resolution of a skin photosensitivity reaction, which is important with the use of photosensitizers. This has enabled the period of necessary light shielding in a room with measurement of ≦ 500 lux to be reduced to 2 weeks for TPS, whereas porfimer sodium requires patients to stay in a semi-dark (≦ 300 lux) room for 1 month after administration. A diode laser instrument, a compact system weighing 14 kg, has low power consumption, and can easily be maintained without the need for dye exchange.

### Operating Microscope Equipped With Diode Laser (Figure 1)

The PD Laser®(Panasonic HealthCare Co., Ltd.), installed in the MM30 microscope (Mitaka Kohki Co., Ltd., Tokyo, Japan), is combined into a system that provides laser irradiation (664 nm) from the operating microscope from a plane nearly coaxial to the surgical view. The laser light is introduced into the microscope by a quartz fiber, and provides a laser transmission path close to an observation light path using a conventional halogen light. This allows surgeons to accurately identify an irradiation target area during surgery. The wavelength of fluorescence from tumor tissues is 672 nm. To detect this fluorescence, a system was established using a very narrow notch filter (Nitto Optical Co., Ltd., Tokyo, Japan) that can capture an 8-nm difference. The system gathers emitted fluorescence through a cooled charge-coupled device (CCD) and visualizes images on the monitor. The system is designed for enhancing the contrast between the brain surface and the tumor, by mounting 6 white light-emitting diodes (LEDs) as light sources (NSPW500BS and NSPW510BS, Nichia Corporation, Tokushima, Japan) around the objective lens to achieve a clear fluorescence observation by brain surface illumination with LEDs allowing 6-level light intensity control (Figures 1C–F).

### Phantom Experiment

We conducted an experiment to examine whether this microscopic system allows appropriate intraoperative fluorescence observation during brain tumor surgery. TPS at 3 concentrations of 1, 10, and 100 μg/mL each was mixed with 10% bovine serum albumin (BSA; Wako Pure Chemical Industries Ltd., Osaka, Japan). Then, a cotton ball was moistened with each mixture. These cotton balls were irradiated with a diode laser at 664 nm under the irradiation conditions described later, and then observed the emitted fluorescence at 672 nm using the microscopic system to determine whether the dose-dependent fluorescence intensity is grossly detectable.

### Subjects

The study subjects were 47 consecutive patients who received the protocol-specified surgery after being diagnosed with glioma by preoperative diagnostic imaging by a single surgeon (JA) from April 2005 to December 2008 at the Department of Neurosurgery, Tokyo Medical University. The final pathological diagnosis was glioma in 42 patients, newly diagnosed tumor in 24 patients, and recurrent tumor in 18 patients. The histological malignancy grade was Grade I in 1 patient, Grade II in 5 patients, Grade III in 8 patients and Grade IV in 28 patients. There were 5 patients whose final pathological diagnosis was not glioma after undergoing the protocol-specified surgery based on the diagnosis of glioma made by preoperative diagnostic imaging. The final pathological diagnosis in these patients was metastatic brain tumor in 2 patients (from lung cancer and mammary gland cancer), primary central nervous system lymphoma in 1 patient and meningioma in 2 patients. The institutional review board approved the participation of humans in research at Tokyo Medical University approved our fluorescence-guided intracranial tumor resection protocol, and all patients gave their informed consent before participating.

### PDT and Intraoperative PD Procedures

TPS was administered as a bolus intravenously at a dose of 40 mg/m^2^ in light-shielded conditions 24 h prior to surgery. Craniotomy was performed under illumination at ≤500 lux. First, the brain surface was observed under halogen light illumination. Then, the halogen light was turned off, and the brain surface was irradiated with a diode laser at 664 nm at a power density of 10 mW/cm^2^, with a beam diameter of 40 mm and an irradiation area of 12.6 cm^2^, to observe the presence or absence of tumor fluorescence. The illuminance of the white LEDs was adjusted as appropriate to check the difference in color tone from the brain surface. Every time tumor tissue was resected, the resected tissue was irradiated with a laser in order to check the intensity of the tumor fluorescence. The fluorescence intensity displayed on the monitor was grossly assessed in three grades: strong fluorescence (S), weak fluorescence (W), and no fluorescence (N). The tumor resection was performed using an optical navigation system (Brainlab K.K., Tokyo, Japan), as awake craniotomy and fluorescence-guided resection, under physiological monitoring. When reaching the limit for resection, the resection cavity was irradiated with a diode laser. When weak or strong fluorescence was grossly detected in the cavity, PDT was performed as reported previously (at a power density of 150 mW/cm^2^ and an irradiation energy of 27 J/cm^2^, with a beam diameter of 15 mm and an irradiation area of 1.8 cm^2^), and the operation was ended. After the operation, the patient was managed in a shielded condition under illumination at ≤500 lux, until the result of the skin photosensitivity test turned to negative.

### Measurement of Talaporfin Sodium Concentrations in Brain Tumor Tissue

TPS concentrations were measured according to the method reported by Yoshida et al. (21), using 5-mm cubic tissue blocks of 124 samples from 47 cases excised from each area assessed as strong, weak or no during the surgery. To the resected brain tumor tissue per 100 mg, 5 mL of a mixture of HEPES buffer solution and CH_3_OH (1:9) was added, then the tissue was homogenized while cooling in ice for 1 min, and the supernatant was used as a measurement sample. The TPS and the internal standard, fluoranthene (Wako Pure Chemical Industries Ltd., Osaka, Japan), in each measurement sample were separated based on the principle of reverse phase liquid chromatography (Inertsil® ODS-2, GL Science Inc., Tokyo, Japan), and detected by a fluorescence detector. Based on each peak area obtained, the peak area ratio relative to fluoranthene was calculated and used the values as TPS concentrations. For patients in whom the concentration was measured at multiple sites in each area assessed as strong, weak or no fluorescence, the mean concentration was used for the evaluation.

### Calculation of the Extent Of Resection

Based on MRI images obtained before surgical resection and within 3 days of the resection, the extent of resection was determined. For gadolinium-enhanced tumors, gadolinium-enhanced T1-weighted axial imaging was used. The sum of the products of perpendicular diameters (SPD) of the contrast-enhanced lesions was calculated. Then, the SPD of residual lesions on immediate postoperative imaging was determined, and the extent of resection was calculated. For non-gadolinium-enhanced tumors, the SPD of the areas of prolonged T2 on T2-weighted imaging was assessed to calculate the extent of resection.

### Statistical Analysis

A significance test for TPS concentrations in each tissue was performed by Student's *t*-test, using SPSS analysis software (Advanced Statistics Version 17 by SPSS, Chicago, USA).

## Results

### Phantom Experiment

**Figure 2:**
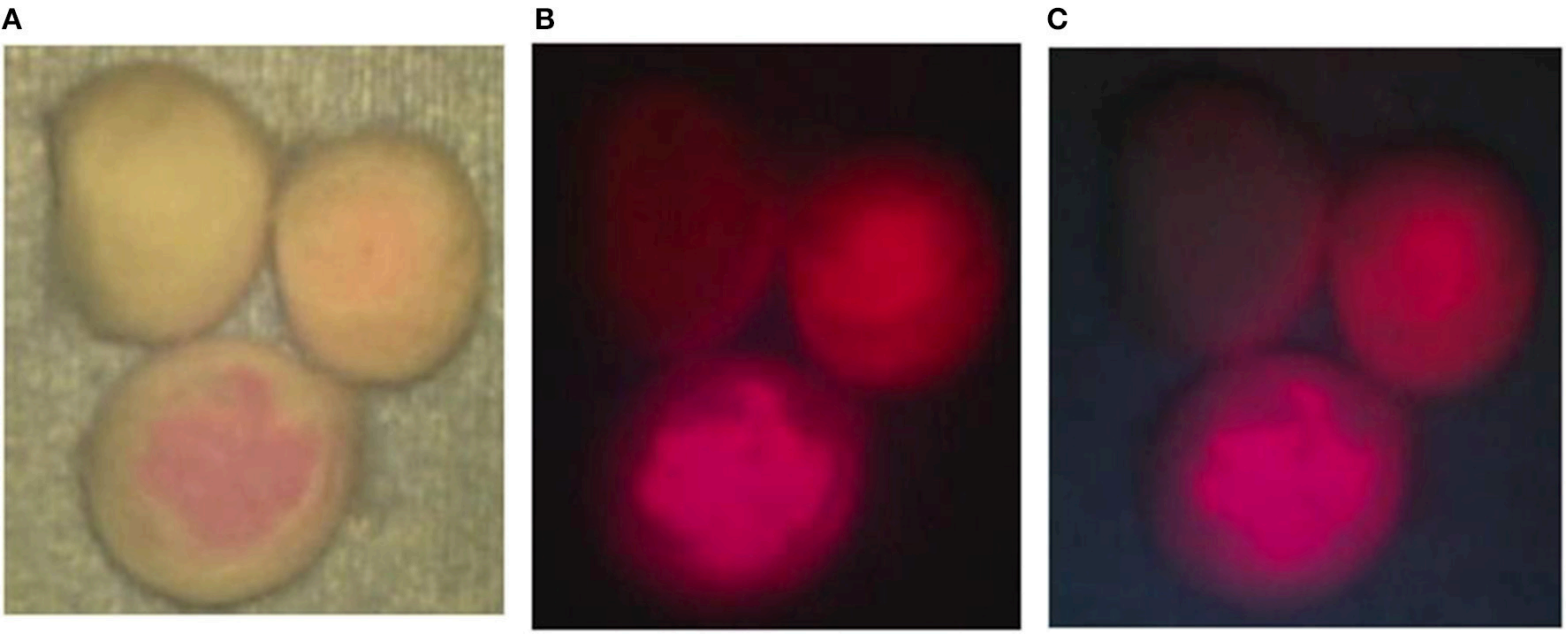
Phantom experiment. **(A)** Talaporfin sodium (TPS) dissolved in 1 mL of physiological saline at 3 concentrations of 1, 10 and 100 μg/mL was mixed with 1 mL of 10% bovine serum albumin (BSA). Then, each mixture was dripped onto a cotton ball. First, when each cotton ball was irradiated with a laser under halogen light illumination of the surgical microscope, clear fluorescence was observed from the cotton ball impregnated with TPS at 100 μg/mL. **(B)** The halogen light illumination of the surgical microscope was turned off, and laser irradiation was performed. Differences in fluorescence intensity dependent on the TPS concentration were clearly identified. **(C)** When laser irradiation was performed while the halogen light was off and the 4 LED light sources were on, color tones of the non-woven fabric on the background were perceived, and differences in concentration among the cotton balls became more distinct.

From the cotton balls impregnated with mixtures of the TPS solutions at 3 concentrations and 10% BSA, red fluorescence with intensities dependent on the concentration of TPS was observed by laser irradiation. A clear fluorescence was observed in light shielded conditions. However, in the case of laser irradiation under halogen light illumination, red fluorescence was visible only from the tissue containing TPS at the concentration of 100 μg/mL, but fluorescence was hardly perceived at other concentrations. In the case of laser irradiation under white LED illumination, both the dose-dependent red fluorescence at all concentrations of TPS and the background are considered observable (Figures 2A–C).

### Representative Cases

**Figure 3:**
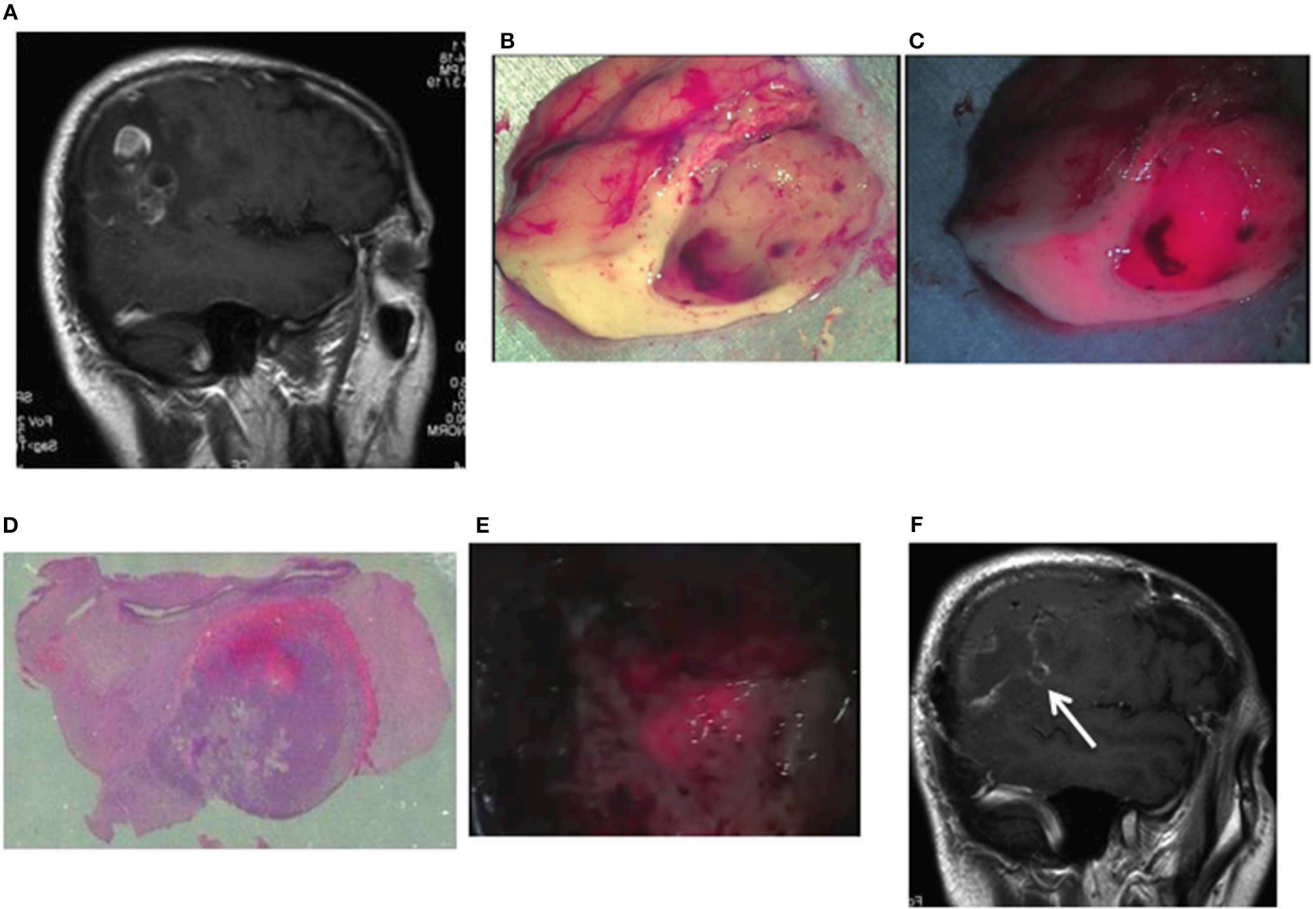
Representative case 10. **(A)** A gadolinium-enhanced T1-weighted sagittal image. The image revealed an irregularly enhanced glioblastoma lesion in the right parietal lobe. **(B)** Brain tissues containing a tumor tissue were resected to the widest extent possible, using an optical navigation system while somatosensory-evoked monitoring was performed. Well-demarcated tumor tissue was observed in the subcortical white matter. **(C)** When laser irradiation was performed under LED illumination, strong fluorescence from the tumor bulk and weak fluorescence from the surrounding white matter were observed. **(D)** A postoperative histopathological image revealed the presence of tumor cells infiltrating from the strong fluorescence area into the weak fluorescence area (hematoxylin and eosin staining). **(E)** When a laser was irradiated to the white matter in the tumor resection cavity under LED illumination, weak fluorescence areas were found in the normal white matter. Therefore, the tissue resection was continued until the fluorescence disappeared. **(F)** The postoperative gadolinium-enhanced MRI confirmed the additionally resected areas (arrow) as well as the total resection of the enhanced lesion.

Case 10: A 56-year-old man had glioblastoma in the right parietal lobe, manifested by involuntary twitching at the left corner of the mouth. The tumor was resected en bloc using an optical navigation system under continuous somatosensory evoked potential monitoring. Being irradiated with a laser, resected tissues emitted strong red fluorescence, with weak red fluorescence in the surrounding area. The TPS concentration in tissue was 2.9538 μg/g in the area of strong fluorescence and 1.5765 μg/g in the area of weak fluorescence. The area of strong fluorescence was within the tumor bulk, and the area of weak fluorescence was within the surrounding brain tissues infiltrated with tumor cells. When the resection cavity was observed under laser irradiation, an area of weak fluorescence was detected and therefore was additionally resected. Pathologically, this area was assessed as a tumor infiltration area containing MIB-1 positive cells. A postoperative contrast-enhanced MRI revealed that the tumor was totally resected, and the additionally resected area was clearly identifiable (Figures 3A–F).

**Figure 4:**
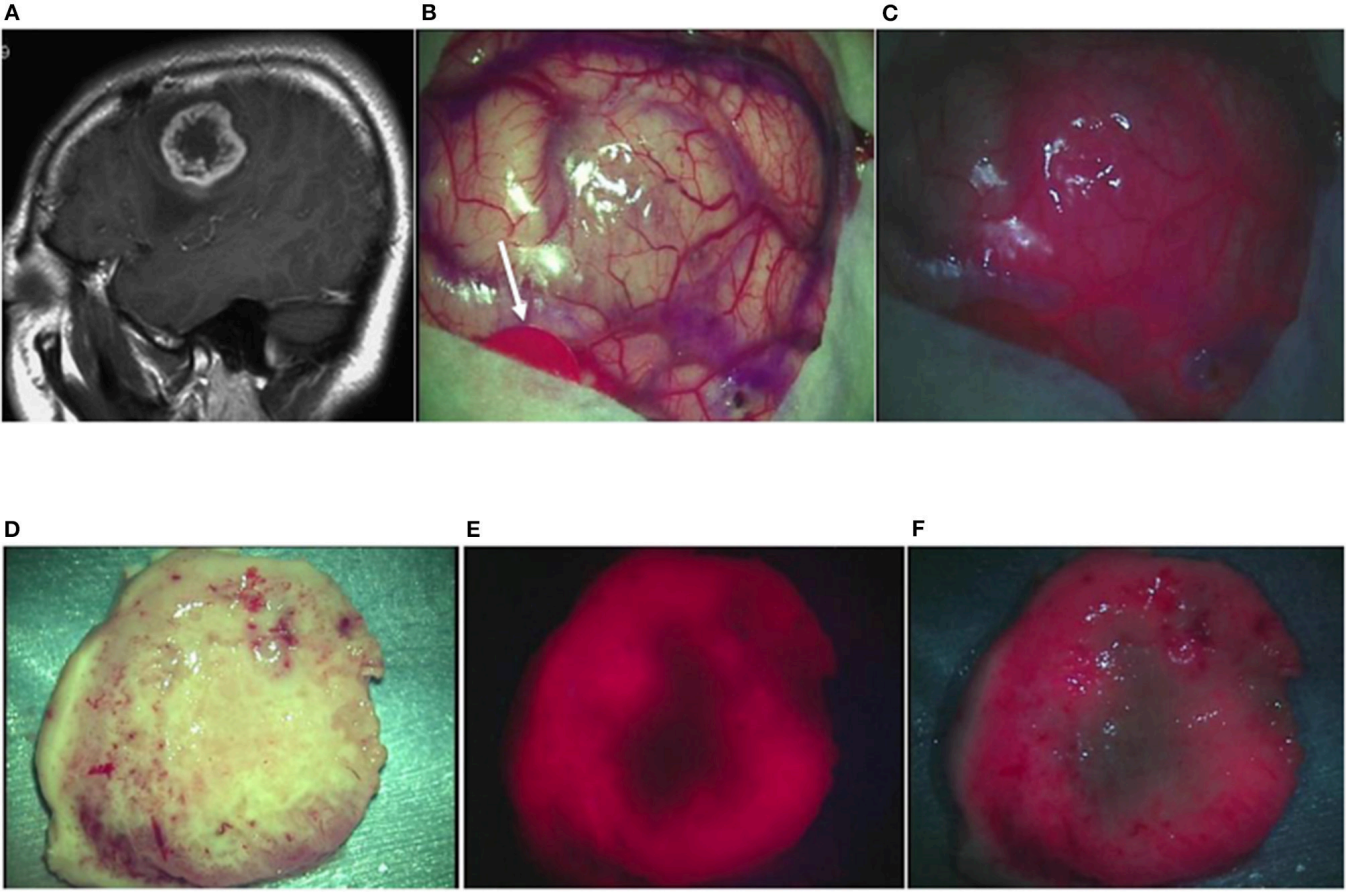
Representative case 18. **(A)** A gadolinium-enhanced T1-weighted sagittal image revealed a ring-like enhanced glioblastoma in the left middle frontal gyrus. **(B)** An image of the brain surface under halogen light illumination showed marked swelling in the left middle frontal gyrus, but tumor tissue was not exposed (arrowed red circle: primary motor cortex). **(C)** In the fluorescence diagnosis under white LED illumination, strong fluorescence from the tumor was observed, and fluorescence from residual TPS in the surrounding blood vessels was identified simultaneously, facilitating the fluorescence-guided resection. **(D)** The tumor together with some surrounding brain tissue attached to the tumor was resected en bloc. The median section observed under halogen light illumination revealed the presence of a light brownish tumor tissue in a doughnut shape. **(E)** Laser irradiation revealed strong fluorescence almost surrounding the central necrotic lesion, clearly identifying the contrast-enhanced lesion on preoperative MRI. **(F)** In the fluorescence diagnosis under white LED illumination, the difference in fluorescence intensity between the strong fluorescence areas and the necrotic lesion or the surrounding brain tissue became more distinct.

Case 18: A 41-year-old woman had glioblastoma in the left frontal lobe, manifested by mild motor aphasia and right hemiplegia. She underwent awake surgery, and laser irradiation was performed on the brain surface during the operation. By the laser irradiation, strong red fluorescence suggestive of a localized tumor was observed on the brain surface along with fluorescence from the blood vessels on the brain surface. When a laser was irradiated under white LEDs, a clear contrast was observed between the fluorescence and the surrounding brain surface. The tumor was resected en bloc and examined on the longitudinal cross-section. Strong ring-like red fluorescence was observed, which was similar to the ring-like enhancement surrounding the central necrosis on MRI images. Observation under LED illumination revealed a more detailed relationship with the surrounding brain tissue. The TPS concentration in tissue was 2.1861 μg/g in the strong fluorescence area, 0.9349 μg/g in the weak fluorescence area, and 0.4044 μg/g in the no fluorescence area in the periphery. A postoperative MRI confirmed that the contrast-enhanced lesion was totally resected (Figures 4A–F).

**Figure 5:**
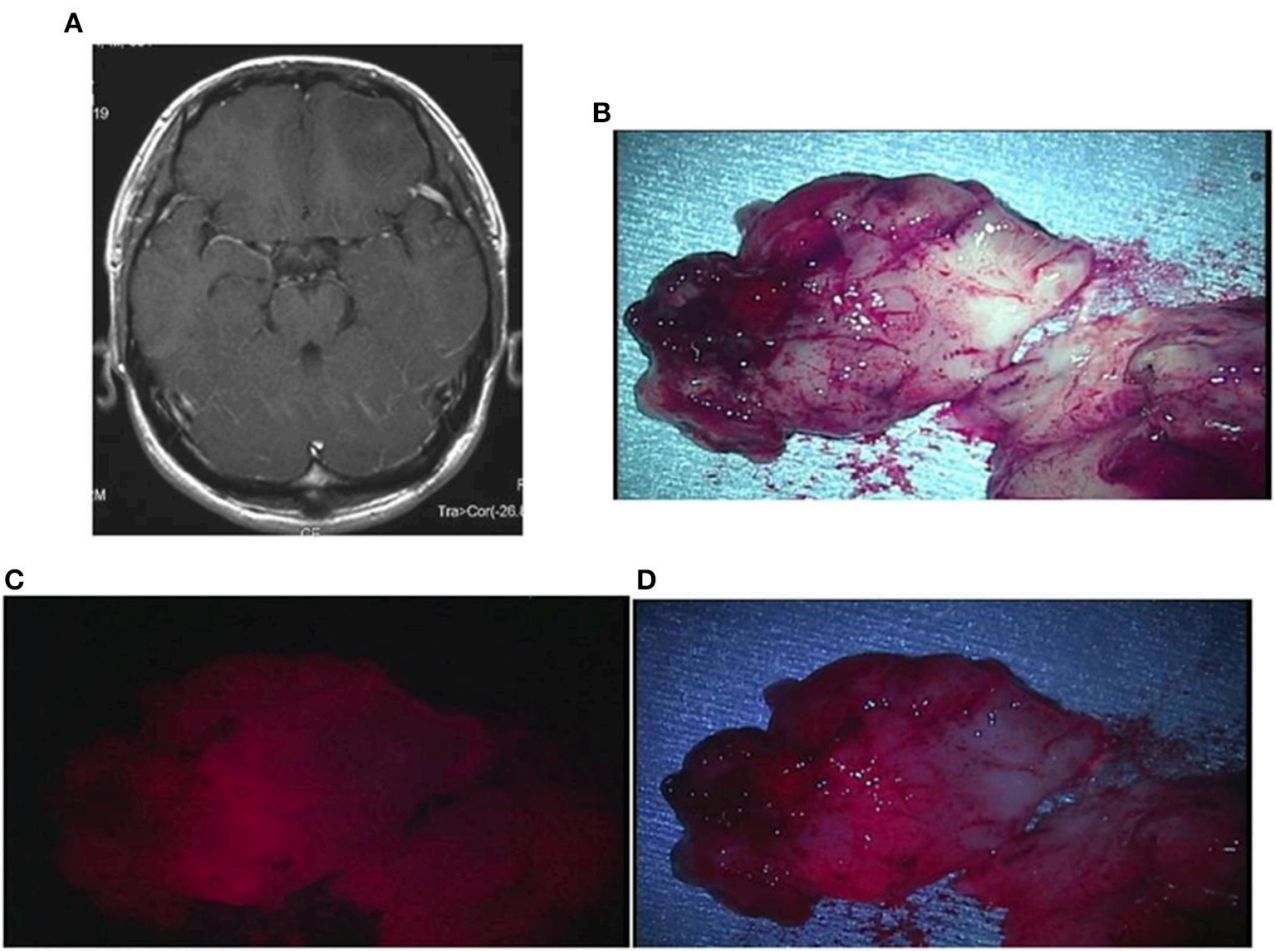
Representative case 2. **(A)** A gadolinium-enhanced T1-weighted axial image showed a slightly enhanced tumor in the left frontal lobe. **(B)** The tumor bulk was resected en bloc. The median section observed under halogen light illumination revealed the presence of a yellowish-gray gelatinous tumor in the cortex. **(C)** By laser irradiation, weak fluorescence from the cortex was detected, and the white matter tended to emit further weaker fluorescence. **(D)** Observed under white LED illumination, the fluorescence from the cortex was enhanced, making the gradation of fluorescence intensity between the cortex and the white matter more distinct.

Case 2: A 30-year-old man had oligoastrocytoma in the left frontal lobe, manifested by a first episode of generalized tonic-clonic seizures. No obvious contrast enhancement was observed on the preoperative contrast-enhanced MRI. When the resected tumor tissue was irradiated with a laser, weak red fluorescence was observed at the site where the tumor had been located. The TPS concentration in tissue in this area was 0.6914 μg/g. A postoperative MRI confirmed that the lesion of prolonged T2 was totally resected (Figures 5A–D).

**Figure 6:**
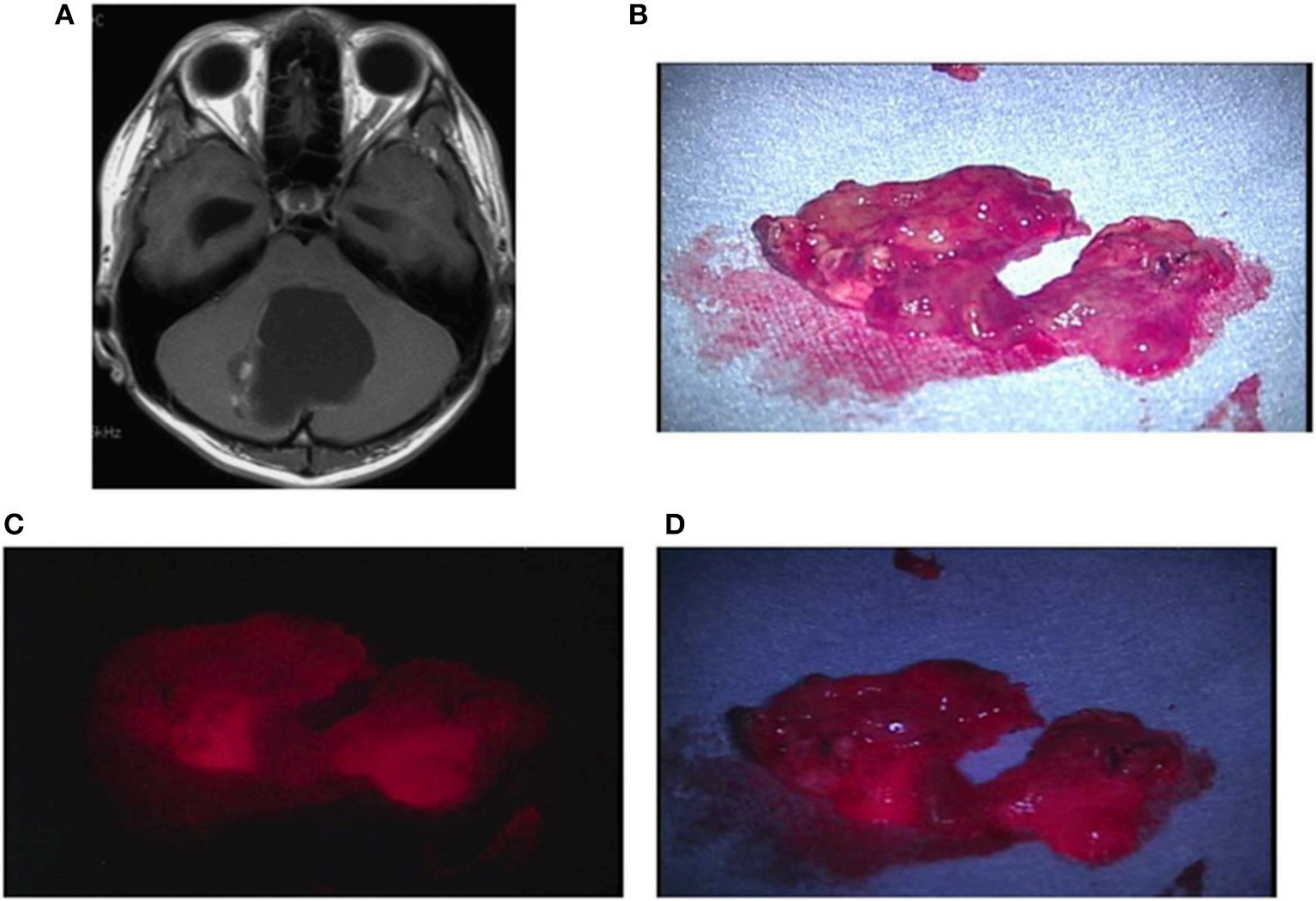
Representative case 1. **(A)** A gadolinium-enhanced T1-weighted axial image showed a cystic tumor with a mural nodule in the vermis cerebelli, in which the nodule was slightly enhanced. **(B)** The resected nodule was rich in blood vessels, exhibiting red color also under halogen light illumination. **(C)** By laser irradiation, strong fluorescence from the nodule was observed. **(D)** Under white LED illumination, strong fluorescence emitted from the entire nodule was clearly observed.

Case 1: An 18-year-old man had pilocytic astrocytoma in the vermis cerebelli, manifested by sudden headache and nausea. A preoperative contrast-enhanced MRI showed an enhanced mural nodule. During the surgery, the cyst was opened and irradiated with a laser. As a result, nodular fluorescence, tending to be strong, appeared with weak fluorescence from the surrounding cystic wall. The TPS concentration in tissue was high, being 3.163 μg/g in the strong fluorescence area and 1.614 μg/g in the weak fluorescence area. A postoperative MRI confirmed that the lesion including the cystic wall was totally resected (Figures 6A–D).

### Results of Intraoperative Observation of Fluorescence From Tumors

#### Fluorescence Positive Rate (Figure 7, Table 1 and Supplementary Table 1)

In glioma cases, 1 case of Grade I pilocytic astrocytoma showed strong fluorescence. In five Grade II cases, none showed strong fluorescence, but all showed weak fluorescence. In eight Grade III cases, all showed at least weak fluorescence, including 5 cases (62.5%) showing strong fluorescence. In 28 Grade IV cases, 22 cases (78.6%) showed strong fluorescence and 26 cases (92.9%) showed weak fluorescence; thus all showed fluorescence, as seen in Grade III cases. In the Grade IV cases, strong fluorescence was shown in 14 (93.3%) of 15 newly diagnosed cases and 8 (61.5%) of 13 recurrent cases. In the recurrent cases, some showed only weak fluorescence. In the other 5 cases, except for 1 case of metastatic brain tumor, 4 cases showed strong fluorescence.

**Figure 7:**
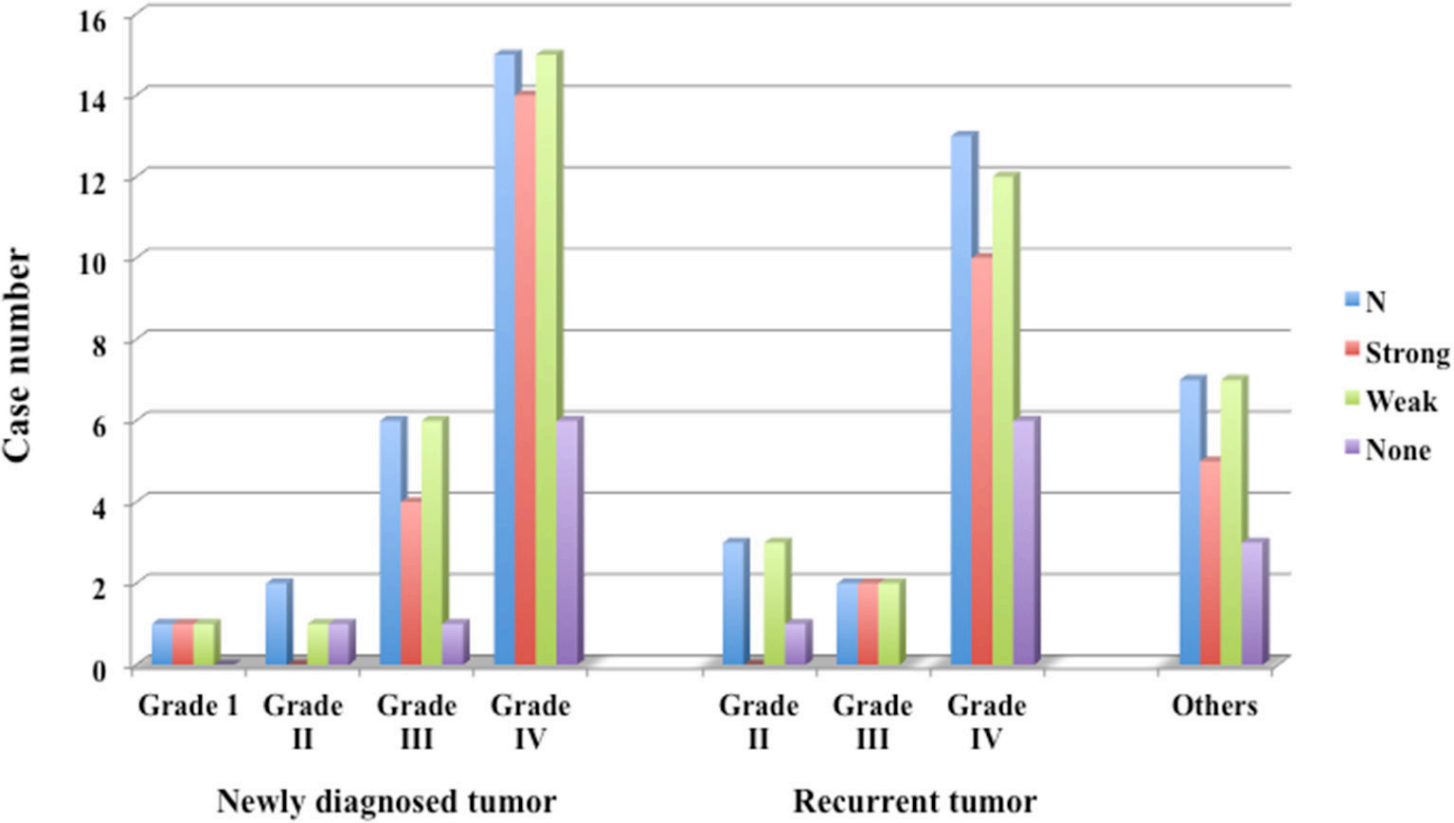
Fluorescence positive rate of newly diagnosed glioma, recurrent glioma and other tumors. In Grade IV, 92.9% of newly diagnosed cases and 61.5% of recurrent cases emitted strong fluorescence.

**Table 1:** Pathological diagnosis and fluorescence positive rate, tissue TPS concentrations, extent of resection and number of patients who underwent PDT.

	**N**	**Fluorescence**			**Concentration (μg/g)**			**Resection**		**PDT**
		**Strong**	**Weak**	**None**	**Strong**	**Weak**	**None**	**N of GTR**	**EOR**	***N***
Newly diagnosed tumor	24									
Grade I	1	1	1	0	3.163	1.614		1	100%	0
Grade II	2	0	2	1		0.489 ± 0.145	0.123	1	95% (95–100)	0
Grade III	6	3	6	3	1.573 ± 0.901	0.608 ± 0.304	0.2	3	98% (90–100)	1
Grade IV	15	14	15	6	1.580 ± 0.908	0.675 ± 0.413	0.193 ± 0.156	11	98% (89–100)	4
Recurrent tumor	18									
Grade I	0									
Grade II	3	0	3	1		0.878 ± 0.276	0.46	1	92% (80–100)	1
Grade III	2	2	2	0	1.112 ± 0.529	0.776 ± 0.375		0	88% (80–95)	1
Grade IV	13	8	11	6	1.347 ± 0.524	0.656 ± 0.381	0.132 ± 0.156	5	88% (63–100)	9
Others										
Meta	2	1	2	1	0.851	0.520 ± 0.097		2	100%	0
PCNSL	1	1	1		4.166 ± 0.294	0.647		1	100%	0
MGM	1	1	1	1	1.657 ± 0.735	0.8382 ± 0.807	0.279	1	100%	0
Atypical MGM	1	1	1	1	1.103 ± 0.957	0.720 ± 0.390	0.424	0	75%	1

*N, number; Meta, metastatic brain tumor; PCNSL, primary central nervous system lymphoma; MGM, meningioma; GTR, gross total removal; EOR, extent of resection; PDT, photodynamic therapy*.

#### Correlation Between Fluorescence Intensity and Tissue Concentration (Figure 8, Table 1 and Supplementary Table 1)

The TPS concentrations in tissue in the strong, weak and no fluorescence areas that were assessed grossly were 1.6184 ± 0.9661, 0.6708 ± 0.3765 and 0.1885 ± 0.1253 μg/g, respectively. There were significant differences in concentration between the strong and weak fluorescence areas and between the weak and no fluorescence areas (*P* < 0.001). In the glioma cases, 1 Grade I case showed strong fluorescence with a tissue concentration of 3.1628 μg/g, but all Grade II cases showed weak fluorescence. The tissue concentrations in strong fluorescence areas in Grade III and IV cases were 1.3751 ± 0.7480 and 1.4948 ± 0.7783 μg/g, respectively, showing no significant difference between the Grade III and IV cases (*P* = 0.718). In Grade IV cases, the mean tissue concentrations in strong, weak and no fluorescence areas were 1.4948, 0.6752, and 0.1655 μg/g, respectively, showing significant differences (*P* < 0.001) among the tissue concentrations in these areas. The tissue concentration in the strong fluorescence area in Grade IV cases was 1.5797 ± 0.9082 μg/g in newly diagnosed cases and 1.3589 ± 0.5239 μg/g in recurrent cases, showing no significant difference between the newly diagnosed and recurrent cases (*P* = 0.493). Also in the weak and no fluorescence areas, no significant differences were observed between the newly diagnosed and recurrent cases (*P* = 0.821 for weak fluorescence area and *P* = 0.853 for no fluorescence area). In non-glioma cases, the tissue concentration in the strong fluorescence area was 2.100 ± 1.528 μg/g, showing no significant difference from the result obtained for the strong fluorescence area in Grade IV cases (*P* = 0.153).

**Figure 8:**
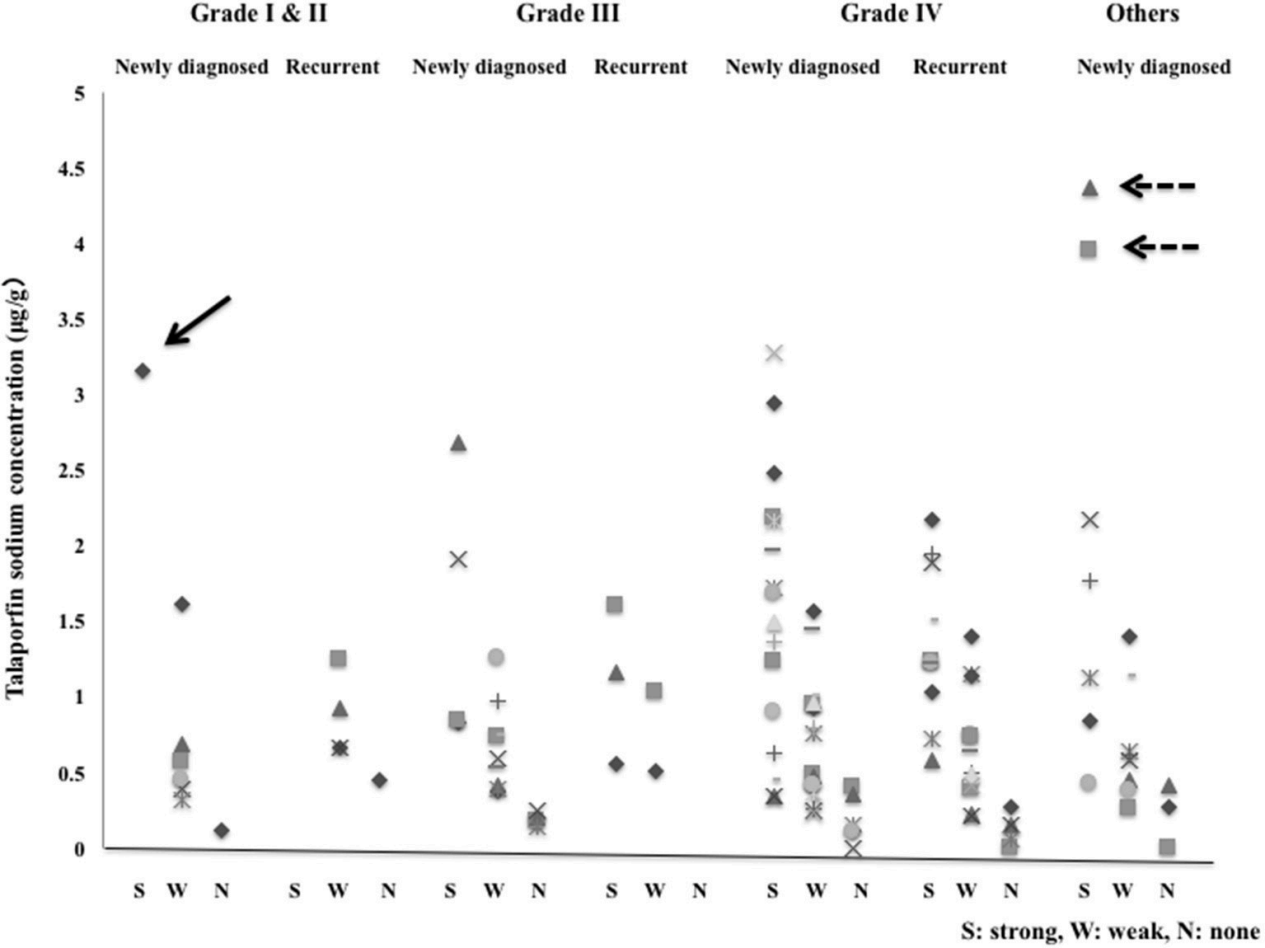
The tissue concentrations of talaporfin sodium in the strong fluorescence (S), weak fluorescence (W) and no fluorescence (N) areas in newly diagnosed cases and recurrent cases with Grade I to IV gliomas and other tumors. Arrow: Pilocytic astrocytoma, Dotted arrow: Primary central nervous system lymphoma.

### Extent of Resection (Supplementary Table 1)

By the FGR using TPS, total resection confirmed on imaging was achieved in 22 (52.3%) of 42 patients with gliomas. In most of the 24 newly diagnosed cases, a clear difference in fluorescence intensity was obtained, allowing the resection of not only strong fluorescence areas but also weak to no fluorescence areas. As a result, total resection was achieved in 16 cases (66.7%). Even in cases with residual lesions, the resection of 94 ± 4.3% (89–99%) of the tumor was achieved, according to an SPD analysis. On the other hand, in 18 recurrent cases, it was difficult to obtain a clear difference in fluorescence intensity. In the majority of these cases, weak fluorescence was blurrily spread, making it difficult to grossly determine the disappearance of the fluorescence. Consequently, total resection was achieved only in 6 cases (33.3%), which is an extremely low extent of resection. In cases showing residual lesions, the extent of resection was limited to 81.4 ± 8.9% (63−95%). Of course, when a tumor clearly infiltrates in the functional regions of the brain, it is difficult to resect the tumor even though fluorescence is detected, and PDT should be performed additionally. PDT was performed in 5 newly diagnosed cases and 11 recurrent cases. In most of these recurrent cases, PDT was added due to concern that there may be residual tumors because it was difficult to confirm the disappearance of fluorescence. In non-glioma cases, 1 case with atypical meningioma with repeated recurrences underwent PDT for the residual tumor because total resection had to be abandoned due to intracerebral infiltration of the tumor in the motor cortex, although fluorescence was identified. In this case, however, total resection of other tumor lesions was achieved.

## Discussion

In the surgery of glioma, which is characterized by invasive growth, the concept of “maximal safe resection” is important. Evidence for the fact that enhancement of the extent of surgical resection leads to improved prognosis of malignant gliomas has been accumulating, and there have been a series of discussions regarding how to achieve total resection of gadolinium-enhanced lesions on MRI images and, at the same time, how to protect neurological functions (22–24). FGR using dyes, such as fluorescein and 5-ALA, has been widely accepted as an intraoperative real-time navigation method that visualizes tumor bulk, and many studies on the efficacy of FGR have been reported (25–27). A representative study among these is a randomized controlled trial (RCT) of FGR with 5-ALA, led by Stummer et al. in which FGR has been demonstrated to improve the extent of resection, resulting in significant prolongation of PFS in patients who underwent FGR. Therefore, this methodology has been accepted in many countries (9, 11, 12). However, FGR with 5-ALA alone failed to prolong overall survival (OS). In other words, the results indicate that the total resection of gadolinium-enhanced lesions by FGR only does not prolong OS (10).

A scheme proposed by Wilson for the extent of tumor cell infiltration shows that curative resection cannot be achieved by glioma surgery, as well as that resection of not only contrast-enhanced lesions on imaging but also the surrounding tissue infiltrated by tumor cells can contribute to reducing the residual tumor cells (28). In recent years, a supra-total resection, i.e., an extended resection of high signal-intensity areas on FLAIR images in tissue surrounding the tumor to the extent that the neurological function is not deteriorated, has been reported to prolong OS (29, 30). Therefore, in malignant glioma surgery, in addition to total resection of gadolinium-enhanced lesions using FGR under proper monitoring of neurological functions, the surrounding tissue infiltrated by tumor cells should be resected to the extent possible up to the boundary of the functional regions of the brain (29, 30). For cases where tumor cells have infiltrated in the functional region of the brain, we consider it significant to perform PDT, which is capable of specifically destroying tumor cells (18, 19). In that sense, the TPS we used this time is a PS that can be used in two ways, not only for PD but also for PDT. Thus, TPS is a tool allowing us to carry out the best approach for malignant glioma. In fact, clinical studies of PDT using TPS have reported PFS of 12 months and OS of 24.8 months in patients with newly diagnosed glioblastoma, showing a clear therapeutic add-on effect to the standard treatment, and this method has been rapidly spreading in Japan (19).

In this article, we report that intraoperative PD using TPS and an excitation diode laser can identify gadolinium-enhanced lesions and non-gadolinium-enhanced lesions infiltrated by tumor cells, based on differences in fluorescence intensity, in glioma cases. In particular, a positive correlation was observed between the histological malignancy grade and the fluorescence positive rate or the fluorescence intensity. In particular, in Grade IV cases, strong fluorescence was observed in 93.3% of newly diagnosed cases and 61.5% of recurrent cases, and there were no cases without fluorescence. In recurrent cases, the fluorescence intensity tended to be weaker than that observed in newly diagnosed cases. The reason for this might be associated with the use of radiotherapy in these cases. Even in cases of lower grade gliomas, in which the detection of fluorescence with the use of 5-ALA is difficult, weak fluorescence was observed, although the number of cases was limited. These results suggest the usefulness of FGR using TPS in all glioma surgeries. Very strong fluorescence was observed in 1 case of pilocytic astrocytoma. In addition, strong fluorescence was observed also in cases of malignant lymphoma, metastases and malignant meningioma. These results suggest that FGR using TPS can be performed for tumors enhanced by gadolinium on MRI, regardless of histological type.

Tsurubuchi et al. (31) examined the intracerebral distribution of 5-ALA and TPS, using a glioma rat model, and reported that the lesion-to-normal brain ratio (L/N ratio) in the tumor bulk was 7.78 ± 4.61 at 2 h after administration of 5-ALA and 23.1 ± 11.9 at 12 h after administration of TPS, with the fluorescence intensity being approximately 10-fold stronger with TPS than with 5-ALA (31). On the other hand, both drugs elicited fluorescence even in vasogenic edema in a cold injury model for brain edema. With 5-ALA, the time to peak fluorescence after administration in edema was delayed compared with that in tumor. On the other hand, with TPS, the time to peak fluorescence was 2 h after administration in peritumoral edema and 12 h after administration in tumor tissue. This time course of the fluorescence intensity perfectly reflects the mechanism of TPS uptake by tumor cells, which is considered as follows: TPS, a water-soluble drug, is conjugated with albumin immediately after intravenous injection, and the conjugate circulates in the bloodstream. After leaking from tumor blood vessels due to the disruption of the blood brain barrier (BBB), the conjugate is taken up by tumor cells, mediated by SLC46A1, a heme carrier protein 1, and other factors, and accumulates into the tumor cells due to the enhanced permeability and retention (EPR) effect (31, 32). Comparing the distribution of fluorescence intensity in resected tissues with the images in the clinical cases presented this time, it was confirmed that the gadolinium-enhanced lesions on MRI exhibited strong fluorescence, with high TPS concentrations in these tissues, suggesting that these findings reflect the TPS uptake by tumor cells. In addition, the findings highly suggest that the weak fluorescence in non-enhanced areas is derived from not only the TPS taken up by infiltrating tumor cells but also the TPS diffused extracellularly. Also in the areas grossly assessed as showing no fluorescence, TPS, which is not present in normal brain tissue, was detected although its concentration was low. The TPS detected may have been trace amounts of TPS diffused outside the tumor cells or the TPS circulating in normal cerebral blood vessels. In addition, since peritumoral brain edema is found also in low-grade gliomas, in which the BBB is generally preserved, it is considered that, as the first step, TPS diffused into the edema fluid emits fluorescence after being taken up by the tumor cells. Particularly in oligodendrogliomas, which is associated with a large volume of the tumor vascular bed, the fluorescence observed may be derived from the TPS present in the tumor blood vessels. This inference is reasonable, because strong fluorescence with a high tissue concentration was observed in pilocytic astrocytoma, although only 1 case was examined.

The finding that the fluorescence intensity was dependent on the histological malignancy grade of glioma cells suggests that the fluorescence intensity of TPS reflects the tumor cell density, proliferative capacity or vascular bed volume, as seen in the studies of 5-ALA (33). Therefore, it is essential to examine the relationship of the fluorescene intensity of TPS with histopathological images in terms of these factors (34).

According to the drug information of TPS, the t_1/2α_ and t_1/2β_ of TPS are 14.6 ± 2.96 and 138 ± 21.4 h, respectively (35). Experiments in rats revealed that the TPS concentration in cerebral tissue 24 h after administration of TPS at 40 mg/m^2^ was one forty-fifth of the plasma concentration (4.56 ± 0.65 μg/g in plasma and 0.09 ± 0.01 μg/g in cerebral tissue). In humans receiving an intravenous injection of TPS at 40 mg/m^2^, the plasma TPS concentration 24 h after administration was 11 μg/g. The TPS concentration in normal cerebral tissue 24 h after administration of TPS at 40 mg/m^2^ is calculated to be 0.24 μg/g. On the other hand, the mean TPS concentration in no fluorescence areas measured by the methodology presented in this article was 0.21 μg/g. Since this measured value is very close to the calculated value, this method for measuring TPS concentrations is judged as appropriate.

By the FGR using TPS, total resection on imaging was achieved in 52.3% of glioma cases. Particularly in newly diagnosed cases, a clear difference in fluorescence among tumor areas was easily identified also under a microscope. As a result, total resection was achieved in 66.7% of the cases, and PDT was properly performed for tumors infiltrating in the functional regions in the brain. On the other hand, in the majority of recurrent cases, weak fluorescence appeared blurrily, and even though the resection was continued, the fluorescence persisted. The resection was extended to the boundary of the functional regions in the brain and then PDT was implemented in many cases. The reasons for this may include tumor vascular changes due to radiotherapy and decreased proliferative capacity of recurrent tumors. In fact, TPS concentrations in tumors tended to be lower in recurrent cases than in newly diagnosed cases, although there was no significant difference. This issue also needs to be studied in future.

Yoshida et al. reported 0.36- to 5.69-fold higher TPS concentrations in cancer tissue than in normal tissue, measured 4 h after the administration of TPS at 40 mg/m^2^ in 16 patients with early head and neck cancer (21). In addition, they stated, from their experience with PDT performed in these cases, that the TPS concentration in the irradiation target tissue needed to be at least 1 μg/g in order to demonstrate the efficacy of PDT (21). In the present study, the mean TPS concentration in tumor tissue 24 h after the administration of TPS in glioblastoma cases was 1.580 μg/g in strong fluorescence areas and 0.672 μg/g in weak fluorescence areas. The tissue TPS concentration in strong fluorescence areas was 7.52-fold higher than that in normal brain tissue. Although there are differences in the time after the administration of TPS to observation as well as in the PDT implementation conditions, these findings suggest that PDT may not have clinical significance in some cases because, unless the target tissue emits at least weak fluorescence, reactions to PDT are unlikely to occur. This needs to be kept in mind when considering the implementation of PDT after FGR in malignant glioma cases in the future.

There are several limitations to acknowledge in this study. First, since this case group was enrolled only for a certain period of time, it can not deny that there was a limit in the number of cases. In particular, as TPS is a photosensitizer that is used for PDT, which is generally only performed on patients who are suspected of having a malignant brain tumor on preoperative neuroimaging, there is little data on the use of TPS in brain tumors except glioma. Second, there was lack of investigation of relationship between histopathological findings and fluorescence intensity or TPS concentration. Such analysis is the focus of our ongoing study.

## Conclusion

We examined the fluorescence emission from malignant brain tumors, using TPS, a second-generation photosensitizer, and an excitation laser specific to it, and demonstrated that TPS is applicable to FGR. In glioma cases, the fluorescence intensity and the TPS concentration in tumor tissue have been found to be correlated with the histological malignancy grade, and, particularly in newly diagnosed patients, have been suggested to contribute to improving the extent of resection. We will further investigate the relationship between histopathological images of brain tumors and the fluorescence intensity or tissue TPS concentration, in order to verify the usefulness of FGR using TPS in malignant brain tumor surgery.

## Author Contributions

MK and AM supervised the project. JA, SF, and MI developed the concept and the design of the study. JA and SF designed and made the experimental set up for data acquisition. JA, SF, and MI carried out patient care, surgery, and sample acquisition. JA performed data collection. JA, SF, MI, AM, and MK analyzed and interpreted the data. JA drafted the manuscript. SF and MK critically revised the manuscript. AM was contributed to the review of the manuscript and answer to the reviewer's comment. All authors have approved the final manuscript.

### Conflict of Interest Statement

The authors declare that the research was conducted in the absence of any commercial or financial relationships that could be construed as a potential conflict of interest.

## Supplementary Material

Supplementary Table 1 Clinical profile, intensity of emitted fluorescence and tissue concentration of TPS of all cases.

## References

[1] Perria C Capuzzo T Cavagnaro G Datti R Francaviglia N Rivano C First attempts at the photodynamic treatment of human gliomas. J Neurosurg Sci. (1980) 24:119–29. 6267229

[2] Kaye AH Morstyn G Appuzo MJ. Photoradiation therapy and its potential in the management of neurological tumors. J Neurosurg. (1988) 69:1–14. 10.3171/jns.1988.69.1.0001 3288722

[3] Stylli SS Kaye AH MacGregor L Howes M Rajendra P. Photodynamic therapy of high grade glioma- long term survival. J Clin Neurosci. (2005) 12:389–98. 10.1016/j.jocn.2005.01.006 15925768

[4] Muller PJ Wilson BC Photodynamic therapy of brain tumor- a work in progress. Laser Surg Med. (2006) 38: 384–89. 10.1002/lsm.20338 16788926

[5] Eljamel MS. Brain photodiagnosis (PD), fluorescence guided resection (FGR) and photodynamic therapy (PDT): past, present and future. Photodiagnosis Photodyn Ther. (2008) 5:29–35. 10.1016/j.pdpdt.2008.01.006 19356633

[6] Nelson JS Liaw LH Orenstein A Berns MW Mechanism of tumor destruction following photodynamic therapy with hematoporphyrin derivative, chlorine, and phthalocyanine. J Natl Cancer Inst. (1988) 80:1599–605. 10.1093/jnci/80.20.1599 2973528

[7] Castano AP Demidova TN Hamblin MR Mechanism in photodynamic therapy: part one – photosensitizers, photochemistry and cellular localization. Photodiagnosis Photodyn Ther. (2004) 1:279–93. 10.1016/S1572-1000(05)00007-4 25048432 PMC4108220

[8] Stummer W Stocker S Wagner S Stepp H Fritsch C Goetz C. Intraoperative detection of malignant gliomas by 5-aminolevulinic acid-induced prophyrin fluorescence. Neurosurgery. (1988) 42:518–26. 9526986 10.1097/00006123-199803000-00017

[9] Stummer W Novotny A Stepp H Goetz C Bise K Reulen HJ. Fluorescence-guided resection of glioblastoma multiforme by using 5-aminolevulinic acid-induced porphyrins: a prospective study in 52 consecutive patients. J Neurosurg. (2000) 93:1003–13. 10.3171/jns.2000.93.6.1003 11117842

[10] Stummer W Pichlmeier U Meinel T Wiestler OD Zanella F Reulen HJ. Fluorescence-guided surgery with 5-aminolevulinic acid for resection of malignant glioma: a randomised controlled multicentre phase III trial. Lancet Oncol. (2006) 7:392–401. 10.1016/S1470-2045(06)70665-9 16648043

[11] Widhalm G Wolfsberger S Minchev G Woeher A Krssak M Czech T 5-Aminolevulinic acid is a promising marker for detection of anaplastic foci in diffusely infiltrating gliomas with non-significant contrast enhancement. Cancer. (2010) 116:1545–52. 10.1002/cncr.24903 20108311

[12] Valdes PA Leblond F Kim A Harris BT Wilson BC Fan X. Quantitative fluorescence in intracranial tumor: implications for ALA-induced PpIX as an intraoperative biomarker. J Neurosurg. (2011) 115:11–17. 10.3171/2011.2.JNS101451 21438658 PMC3129387

[13] Kennedy JC Pottier RH. Endogeneous protoporphyrin IX, a clinically useful photosensitizer for photodynamic therapy. J Photochem Photobiol B. (1992) 14:275–92. 10.1016/1011-1344(92)85108-7 1403373

[14] Roberts DW Valdes PA Harris BT Fontaine KM Hartov A Fan X Coregistered fluorescence-enhanced tumor resection of malignant glioma: relationship between δ-amino-levulinic acid-induced protoporphyrin IX fluorescence, magnetic resonance imaging enhancement, and neuropathological parameters. J Neurosurg. (2011) 114:595–603. 10.3171/2010.2.JNS091322 20380535 PMC2921008

[15] Tsutsumi M Miki Y Akimoto J Haraoka J Aizawa K Hirano M. Photodynamic therapy with talaporfin sodium induces dose-dependent apoptotic cell death in human glioma cell lines. Photodiagnosis Photodyn Ther. (2013) 10:103–10. 10.1016/j.pdpdt.2012.08.002 23769275

[16] Matsumura H Akimoto J Haraoka J Aizawa K. Uptake and retention of the mono-L-asparthyl chlorine e6 in experimental glioma. Lasers Med Sci. (2008) 23:237–45. 10.1007/s10103-007-0469-3 17703335

[17] Namatame H Akimoto J Matsumura H Haraoka J Aizawa K. Photodynamic therapy of C6-implanted glioma cells in the rat brain employing second-generation photosensitizer Talaporfin sodium. Photodiagnosis Photodyn Ther. (2008) 5:198–209. 10.1016/j.pdpdt.2008.08.001 19356656

[18] Akimoto J Haraoka J Aizawa K. Preliminary clinical report of safety and efficacy of photodynamic therapy using Talaporfin sodium for malignant gliomas. Photodiagnosis Photodyn Ther. (2012) 9:91–99. 10.1016/j.pdpdt.2012.01.001 22594978

[19] Muragaki Y Akimoto J Maruyama T Iseki H Ikuta S Nitta M. Phase II clinical study on intraoperative photodynamic therapy with talaporfin sodium and semiconductor laser in patients with malignant brain tumors. J Neurosurg. (2013) 119:845–52. 10.3171/2013.7.JNS13415 23952800

[20] Usuda J Tsutsui H Honda H Ichinose S Ishizumi T Hirata T. (2007) Photodynamic therapy for lung cancers based on novel photodynamic diagnosis using talaporfin sodium (NPe6) and autofluorescence bronchoscopy. Lung Cancer. 58:317–23. 10.1016/j.lungcan.2007.06.026 17698240

[21] Yoshida T Tokashiki R Ito H Shimizu A Nakamura K Hiramatsu H. Therapeutic effects of a new photosensitizer for photodynamic therapy of early head and neck cancer in relation to tissue concentration. Auris Nasus Larynx. (2008) 35:545–51. 10.1016/j.anl.2007.10.008 18242905

[22] Sanai N Polley MY McDermott MW Parsa AT Berger MS. An extent of resection threshold for newly diagnosed glioblastomas. J Neurosurg. (2011) 115:3–8. 10.3171/2011.2.JNS10998 21417701

[23] Chen L Mao Y. Gross total resection plays a leading role in survival of patients with glioblastoma multiforme. World Neurosurg. (2014) 82:105–7. 10.1016/j.wneu.2014.04.074 24802844

[24] Grabowski MM Recinos PF Nowacki AS Schroeder JL Angelov L Barnett GH. Residual tumor volume versus extent of resection: predictors of survival after surgery of glioblastoma. J Neurosurg. (2014) 121:1115–23. 10.3171/2014.7.JNS132449 25192475

[25] Schwake M Stummer W Suero Molina EJ Wolfeer J. Simultaneous fluorescein sodium and 5-ALA in fluorescence-guided glioma surgery. Acta Neurochir. (2015) 157:877–79. 10.1007/s00701-015-2401-0 25820632 PMC4477944

[26] Liu JTC Meza D Sanai N. Trends in fluorescence image-guided surgery for gliomas. Neurosurgery. (2014) 75:61–71. 10.1227/NEU.0000000000000344 24618801 PMC4062574

[27] Zimmermann A Ritsch-Marte M Kostron H. mTHPC-mediated photodynamic diagnosis of malignant brain tumors. Photochem Photobiol. (2001) 74:611–16. 10.1562/0031-8655(2001)074<0611:MMPDOM>2.0.CO;2 11683042

[28] Wilson CB. Glioblastoma: the past, the present, and the future. Clin Neurosurg. (1992) 38:32–48. 1311227

[29] Yordanova YN Moritz SG Duffau H. Awake surgery for WHO II gliomas within “noneloquent” areas in the left dominant hemisphere: toward a “supratotal” resection. J Neurosurg. (2011) 115:232–39. 10.3171/2011.3.JNS101333 21548750

[30] Li YM Suki D Hess K Sawaya R. The influence of maximum safe resection of glioblastoma on survival in 1229 patients: can we do better than gross-total resection? J Neurosurg. (2016) 124:977–988. 10.3171/2015.5.JNS142087 26495941

[31] Tsurubuchi T Zoboronok A Yamamoto T Nakai K Yoshida F Shirakawa M. The optimization of fluorescence imaging of brain tumor tissue differentiated from brain edema- *In vivo* kinetic study of 5-aminolevulinic acid and talaporfin sodium. Photodiagnosis Photodyn Ther. (2009) 6:19–27. 10.1016/j.pdpdt.2009.03.005 19447368

[32] Takada T Tamura M Yamamoto T Matsui H Matsumura A. Selective accumulation of hematoporphyrin deriveative in glioma through proton-coupled folate transporter SLC46A1. J Clin Biochem Nutr. (2014) 54:26–30. 10.3164/jcbn.13-87 24426187 PMC3882491

[33] Stummer W Tonn JC Goetz C Ullrich W Stepp H Bink A 5-Aminolevulinic acid-derived tumor fluorescence: the diagnostic accuracy of visible fluorescence qualities as corroborated by spectrum and histology and postoperative imaging. Neurosurgery. (2014) 74:310–9. 10.1227/NEU.0000000000000267 24335821 PMC4206350

[34] Mitra S Foster TH. *In vivo* confocal fluorescence imaging of the intratumor distribution of the photosensitizer mono-L-aspartylchlorin-e6. Neoplasia. (2008) 10:429–38. 10.1593/neo.08104 18472960 PMC2373909

[35] Kessel D Pharmacokinetics of N-asparthyl chlorin e6 in cancer patients. J Photochem Photobiol B. (1997) 39:81–3. 10.1016/S1011-1344(96)00009-7 9210325

